# Diagnostic value of “hyperdense consolidation sign” as a characteristic new computed tomography sign of diffuse alveolar hemorrhage

**DOI:** 10.1038/s41598-022-25740-y

**Published:** 2022-12-07

**Authors:** Soma Kumasaka, Yuka Kumasaka, Akiko Jingu, Yoshito Tsushima

**Affiliations:** 1 Department of Diagnostic Radiology and Nuclear Medicine, Gunma University Graduate School of Medicine, 3-39-22 Showa-Machi, Maebashi, Gunma 371-8511 Japan; 2 Department of Diagnostic Radiology, Fujioka General Hospital, 813-1 Nakakurisu, Fujioka, Gunma Japan

**Keywords:** Signs and symptoms, Hypoxia, Respiratory tract diseases

## Abstract

Diffuse alveolar hemorrhage (DAH) is an uncommon but life-threatening condition. Although DAH must be distinguished from other lung diseases, no specific computed tomography (CT) signs of DAH have been reported. This study aimed to evaluate the diagnostic value of “hyperdense consolidation” CT sign. We retrospectively evaluated non-contrast CT findings of 25 DAH patients and age- (≤ 2 years) and sex-matched controls with symptoms of dyspnea and hypoxemia. Two radiologists compared the two groups for the presence of hyperdense consolidation signs in lung parenchyma, defined as consolidation that visually contains areas with higher density than the aorta in the specific narrow window setting (window level = 35 Hounsfield units [HU], width = 80 HU) with a mediastinal filter. The sensitivity, specificity, positive- and negative-predictive values of the hyperdense consolidation sign for detection of DAH were 32.0%, 100%, 100%, and 59.5% with perfect interobserver agreement (к = 1.00). The hyperdense consolidation sign was found to be a highly specific sign for DAH.

## Introduction

Diffuse alveolar hemorrhage (DAH) is an uncommon but life-threatening condition characterized by diffuse intra-alveolar bleeding in patients of varying ages^1,2^. Hemoptysis is a characteristic symptom of DAH; however, it may be absent at the time of admission in ≤ 58% of patients^3^, and it can be absent even if alveolar hemorrhage is severe enough to cause anemia^4^. Symptoms other than hemoptysis are varied and nonspecific, such as dyspnea, cough, and fever^1,5,6^. Although bronchoalveolar lavage (BAL) is often required to recognize DAH and exclude other diseases, such as those caused by infection, it is not always performed due to severe dyspnea and increased risk of re-bleeding^7^.

Radiographic and computed tomography (CT) findings of DAH have been reported as ground-glass opacities or consolidations, which may reflect a patchy, focal, or diffuse alveolar filling process^8–10^. These imaging features are nonspecific and may be absent or variable; therefore, the diagnosis of DAH is occasionally challenging^1,11^. Since proper diagnosis of DAH requires integration of symptoms, blood examination, medical imaging findings, bronchoscopic evaluation, and sometimes pathological examination^12^, any diagnostic method capable of suggesting DAH at an early time point would be desirable.

**Figure 1:**
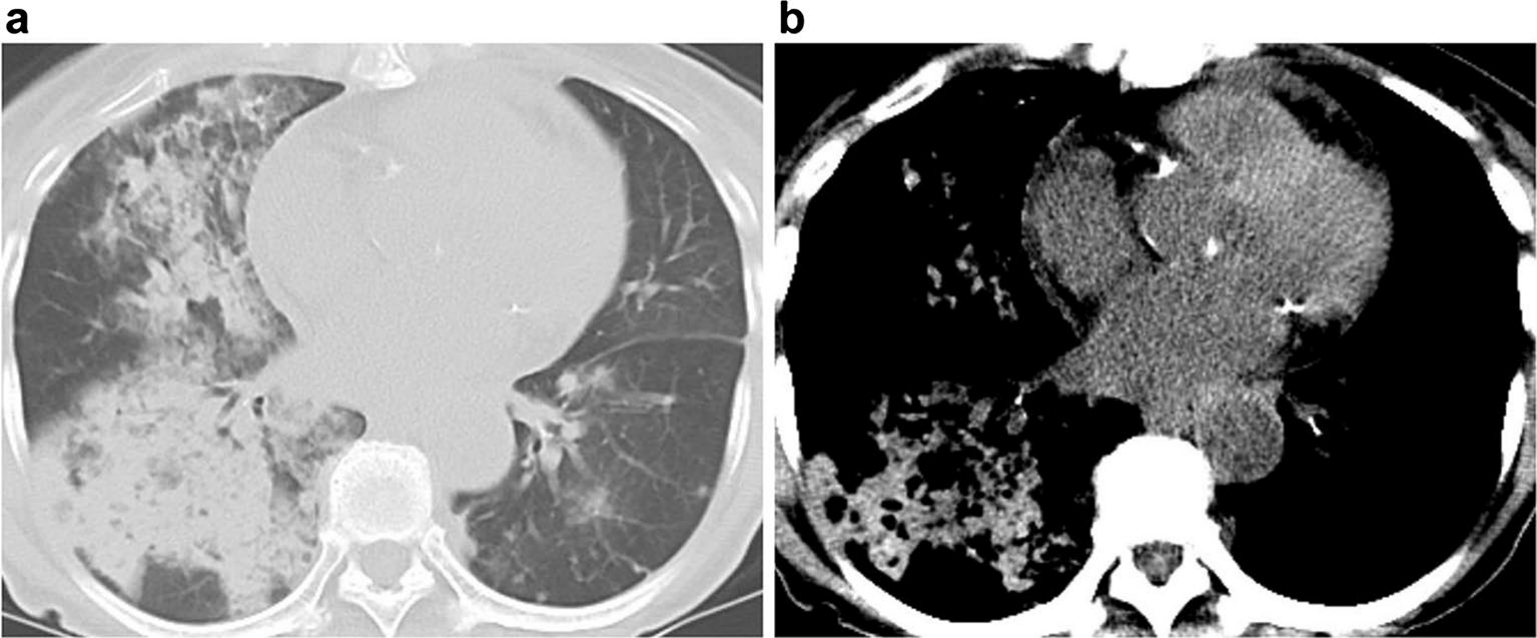
70-year-old woman with diffuse alveolar hemorrhage in microscopic polyangiitis. (**a**) Transaxial CT with a lung window setting showing consolidation in the right lung. (**b**) Hyperdense consolidation is shown with a narrow window setting and a mediastinal filter.

We anecdotally encountered patients with DAH whose chest CT showed consolidations with impressively high density with a narrow window setting (Fig. 1).

On the basis of those CT findings, we speculated that a new CT sign, named the “hyperdense consolidation” sign, could be useful to distinguish DAH from other alveolar filling diseases.

The study aim was to evaluate the diagnostic value of this proposed CT “hyperdense consolidation” sign as an indicator of DAH.

## Materials and methods

### Patients

All DAH patients who presented to one of two hospitals (with 731 beds and 399 beds) between July 2013 and November 2018 were retrospectively evaluated. Diagnosis of DAH was made on the basis of pathological results, bronchoscopic results, or integration of clinical findings and the presence of at least three of four of the following clinical features: hemoptysis, anemia (blood hemoglobin < 10 mg/dl), new alveolar infiltrates on CT, and underlying conditions associated with DAH (vasculitis, capillaritis, anticoagulants, or antiplatelet agents)^6^.

The inclusion criteria were (1) patients whose final diagnosis was DAH, (2) non-contrast-enhanced chest CT in the arms-up position performed ≤ 3 days after appearance of any symptoms related to DAH, and (3) age ≥ 20 years old. Repeated episodes of DAH in a single patient were considered separately. Patients with CT for postmortem imaging, in the arms-down position, or with poor breath holding were excluded from this study.

As a control group, age- (≤ 2 years) and sex-matched patients with symptoms of dyspnea and hypoxemia were randomly selected from our hospital database. Patients with CT showing strong artifacts, in the arms-down position, with poor breath holding, or with pulmonary malignant tumors were excluded. Age, sex, blood hemoglobin, plasma blood urea nitrogen (BUN)/creatinine (Cr) ratio, presence of large-volume fluid administration (> 3000 mL/day) and final diagnosis were recorded for all patients. Underlying diseases were also recorded in patients with DAH.

The study was approved by Gunma University Ethical Review Board for Medical Research Involving Human Subjects (HS2021-137) and was in accordance with the 1964 Helsinki Declaration and its later amendments. Considering the retrospective nature of the study, informed consent was waived, and the opt-out method was employed on the hospital website.

### CT

A multidetector CT scanner equipped with ≥ 64 channels (Aquilion ONE, Canon, Tokyo, Japan; Aquilion 64, Canon, Tokyo, Japan; Lightspeed VCT, GE Medical Systems, Waukesha, WI; Revolution HD, GE Healthcare, Milwaukee, Wisconsin, USA; and SOMATOM Definition Flash, Siemens Healthcare, Forchheim, Germany) was used to perform all CT examinations with automatic exposure control and a tube voltage of 120 kVp. Axial images at 5-mm thickness were used for the following image analysis. The model of CT scanner used in each examination was recorded for all cases.

All CT images of the DAH and control patients were reviewed on a PACS workstation in randomized order by two board-certified diagnostic radiologists separately who were blinded to the diagnoses and other patients’ data. Reader 1 had 10 years and reader 2 had 9 years of experience in CT interpretation. The readers evaluated the images for the presence of hyperdense consolidation signs.

**Figure 2:**
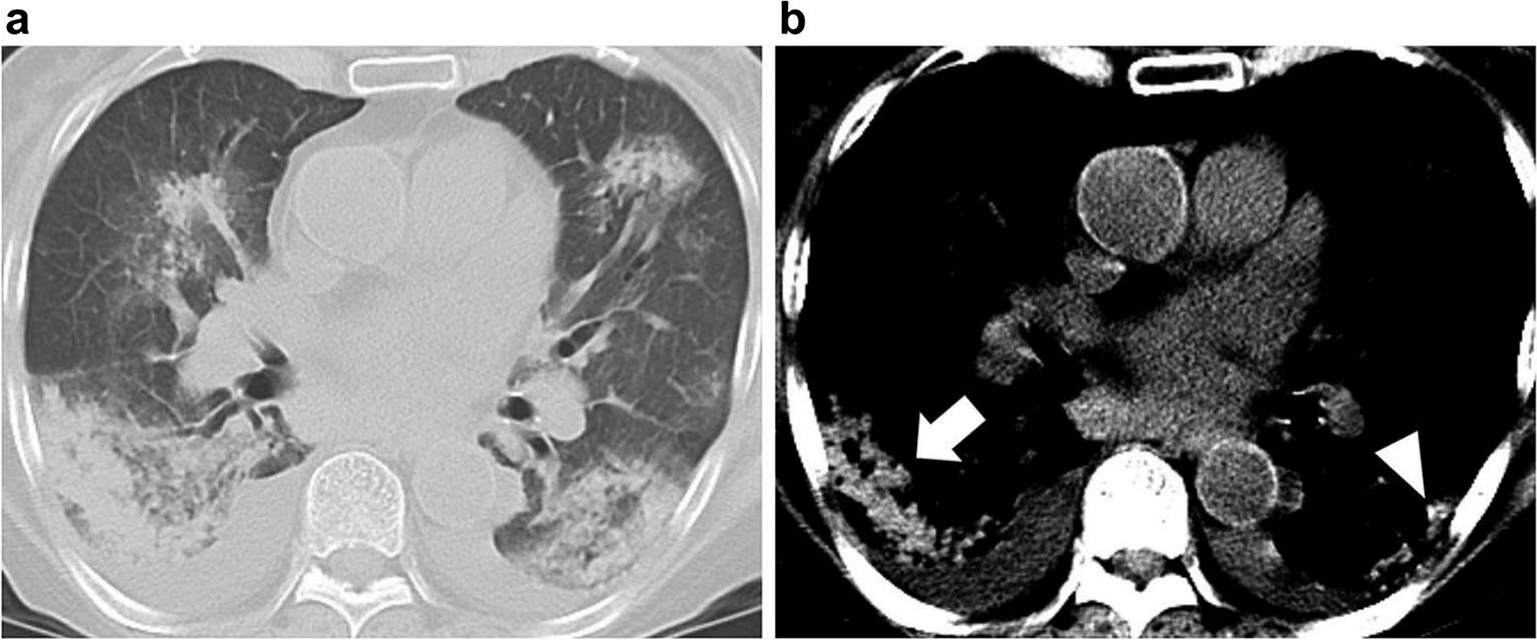
78-year-old woman with diffuse alveolar hemorrhage in Sjögren’s syndrome. (**a**) Transaxial CT with a lung window setting (window level =  − 600 HU, width = 1600 HU) showing consolidations in both lungs. (**b**) In this study, the high-density area in the right lung, which is obviously higher than that of the aorta in the narrow window setting (window level = 35 HU, width = 80 HU), is interpreted as the hyperdense consolidation sign (arrow). On the other hand, the small nodular high-density lesion in the left lung, which can be confused with calcification or artifacts, is interpreted as NOT showing the hyperdense consolidation sign (arrowhead).

The hyperdense consolidation sign was defined as a consolidation visually containing high-density areas obviously higher than the density of the aorta in the specific narrow window setting (window level = 35 Hounsfield units [HU], width = 80 HU) with a mediastinal filter in non-contrast CT. To avoid any potential interobserver conflicts, slightly hyper-density areas were interpreted as NOT being the hyperdense consolidation sign. Small nodular high-density lesions, which can be confused with calcification or artifacts, were also interpreted as NOT showing the hyperdense consolidation sign (Fig. 2).

Both radiologists also reviewed the cases together and evaluated the images for the presence of consolidations, ground-glass opacities, and crazy-paving appearances in the lung window setting (window level =  − 600 HU, width = 1600 HU). Presence of pleural effusion and the CT attenuation value (HU) of the aortic lumen measured from > 100 mm^2^ regions of interest (ROIs) were also recorded.

### Statistical analysis

The sensitivity, specificity, positive predictive value, and negative-predictive value of the hyperdense consolidation sign for the diagnosis of DAH were calculated. Kappa statistics were also used to calculate interobserver agreement for the presence of the hyperdense consolidation sign. We performed the Mann–Whitney *U*-test to compare continuous data and Fisher’s exact test to compare the categorical data between the two groups. The chi-square test was used to compare the five CT scanners for the presence of the hyperdense consolidation sign. All tests were two-sided, and *p* values < 0.05 were considered to be indicative of statistical significance. All statistical analyses were performed in SPSS software (IBM SPSS Statistics 25; IBM Japan, Tokyo, Japan).

### Ethics approval and consent to participate

Approved by Gunma University Ethical Review Board for Medical Research Involving Human Subjects. HS2021-137, 2021/10/12, retrospectively registered. Due to the retrospective nature of the study, informed consent was waived by Gunma University Ethical Review Board for Medical Research Involving Human Subjects, and the opt-out method was employed on the hospital website.

## Results

A total of 39 patients with 43 episodes of DAH were found in the hospital databases. Of these episodes, 18 were excluded because of the absence of non-contrast CT images (n = 5), postmortem imaging (n = 1), age < 20 years old (n = 3), arms-down position (n = 4), or poor breath holding (n = 5). Therefore, a total of 23 patients with 25 episodes of DAH met the study criteria. The time interval between the onset of symptoms and CT scan was 1.0 ± 0.9 days.

The final diagnosis of DAH was established on the basis of clinical findings of hemoptysis (n = 23, 92.0%), anemia (n = 14, 56.0%), new alveolar infiltrates on CT (n = 24, 96.0%), underlying conditions associated with DAH (n = 17, 68.0%), bronchoscopy (n = 8, 32.0%), BAL (n = 5, 20.0%), or pathology (n = 2, 8.0%). The primary etiology of DAH and the final diagnoses of the control patients are summarized in Tables 1 and 2.

**Table 1:** Primary etiology of the patients with DAH.

Primary etiology	Number of episodes (n = 25)
Microscopic polyangiitis	8
Anticoagulants and/or antiplatelet agents	5
Goodpasture’s syndrome	1
Sjögren’s syndrome	1
Anti-phospholipid syndrome	1
Thrombotic microangiopathy	1
Acute myeloid leukemia	1
Idiopathic	7

DAH, diffuse alveolar hemorrhage.

**Table 2:** Final diagnoses of the control patients.

Final diagnoses	Number of episodes (n = 25)
Bacterial pneumonia	10
Pneumocystis pneumonia	5
Acute exacerbation of interstitial lung disease	4
Pulmonary edema	3
Bacterial pneumonia and pulmonary edema	2
Drug-induced pneumonia	1

The hyperdense consolidation sign was positive in 8 of 25 DAH episodes (32.0%) and was not identified in the control group (*p* = 0.004), without statistical significance among the five CT scanners (*p* = 0.131). Out of these hyperdense consolidation sign positive cases (n = 8), only 2 cases had bronchoscopy/BAL confirmations. Perfect agreement between the two radiologists was found for the identification of the hyperdense consolidation sign (к = 1.00). The results of the comparison between two groups are summarized in Table 3.

**Table 3:** Patient characteristics, laboratory data, and CT imaging data.

	DAH group	Control group	*p* value
	(n = 25)	(n = 25)	
Age (years)	64.3 ± 16.7	64.0 ± 16.8	0.961
**Sex**			
Male	16	16	1.000
Female	9	9	
Blood haemoglobin (g/dL)	9.8 ± 3.4	11.5 ± 2.0	0.095
BUN/Cr	20.0 ± 9.3	22.2 ± 10.8	0.347
**Large-volume fluid administration**			
Yes	0	1	1.000
No	25	24	
**Ground-glass opacity**			
Yes	23	17	0.074
No	2	8	
**Crazy-paving appearance**			
Yes	2	4	0.667
No	23	21	
**Consolidation**			
Yes	20	17	0.520
No	5	8	
**Hyperdense consolidation sign**			
Yes	8	0	0.004
No	17	25	
**Pleural effusion**			
Yes	8	17	0.023
No	17	8	
CT attenuation value of aortic lumen (HU)	32.7 ± 9.1	38.2 ± 6.1	0.024

Values are given as n or the mean ± SD.

DAH, diffuse alveolar hemorrhage; BUN, blood urea nitrogen; Cr, creatinine; CT, computed tomography, HU, Hounsfield units.

**Figure 3:**
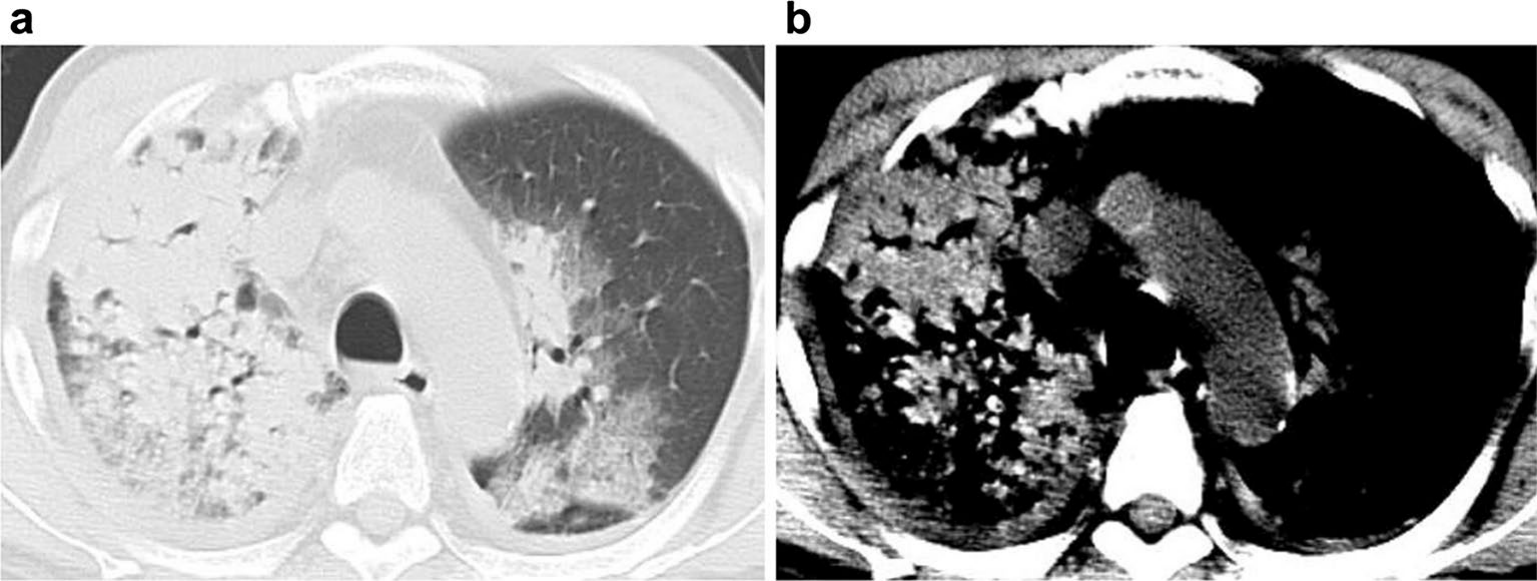
70-year-old man with diffuse alveolar hemorrhage in microscopic polyangiitis. (**a**) Transaxial CT with a lung window setting showing the consolidations and ground-glass opacities. (**b**) The hyperdense consolidation sign is seen in the narrow window setting with a mediastinal filter.

**Figure 4:**
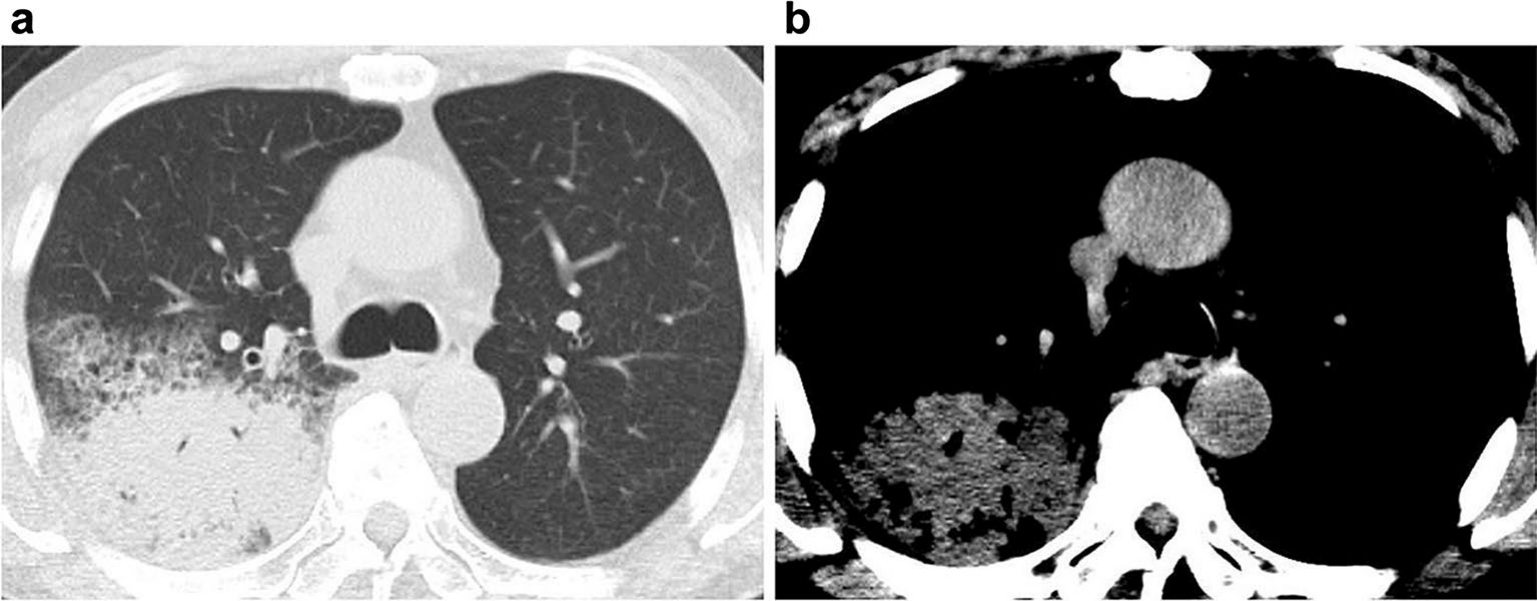
77-year-old man with bacterial pneumonia. (**a**) Transaxial CT with a lung window setting showing consolidations in the right lung. (**b**) In the narrow window setting with a mediastinal filter, the consolidation is iso- to slightly hypodense relative to the density in the aorta, indicating that the findings are negative for the hyperdense consolidation sign.

The sensitivity, specificity, positive-predictive value, and negative-predictive value of the hyperdense consolidation sign for the diagnosis of DAH were 32.0%, 100%, 100%, and 59.5% (Figs. 3, 4).

The absence of pleural effusion and a lower CT attenuation value for the aortic lumen were also associated with DAH patients (*p* = 0.023 and 0.024, respectively). Although blood test results showed a tendency toward lower blood hemoglobin levels in patients with DAH (9.8 ± 3.4 vs. 11.5 ± 2.0 g/dL), the difference was not significant (*p* = 0.095). No significant differences were observed in any other factors.

## Discussion

This is the first study to describe the hyperdense consolidation sign, defined as consolidation with a density that is visually higher than that of the aorta in a specific narrow window setting. Although the hyperdense consolidation sign was not found to be sensitive, it is the only sign with high specificity for distinguishing DAH from other alveolar filling diseases. DAH is a life-threatening condition and must be distinguished from other lung diseases, such as those caused by infections or that cause pulmonary edema. To the best of our knowledge, no specific CT findings of DAH have been reported in the literature, and a specific clinical diagnosis of DAH by CT imaging alone has not been thought possible^12^.

In previous studies of DAH, the evaluation was performed in the regular lung windows^5,9,10,12^. This was not surprising since radiologists typically use regular lung windows when evaluating lung abnormalities, except calcifications, pleural effusion, or pleural thickening. We hypothesized that the narrow mediastinal window setting may enable detection of the slightly higher density of consolidation due to bleeding.

In this study, the CT imaging features of DAH varied from ground-glass opacities to hyperdense consolidation, which were consistent with those of previous reports^8–10^. CT findings of DAH may depend on the level of bleeding and the filling of alveolar spaces. If the alveolar spaces are filled with sufficient bleeding, the lesion can be visualized as hyperdense caused by aggregation of globin molecules, as seen in intracranial hemorrhages^13,14^. Moreover, the CT attenuation value of the aortic lumen may decrease in patients with anemia, which may cause the contrast between the aorta and consolidation and show the latter as hyperdense^15,16^. As expected, there was a significant difference in the CT attenuation values of the aortic lumen between the two groups in this study. Due to clot retraction, the hematocrit may increase to 90% regardless of the baseline hematocrit value of patient's blood; therefore, lung consolidations can be seen as higher density than the aortic lumen on CT^13^. Furthermore, the narrow window setting may make it easier to recognize the slight change in density.

We found that when a hyperdense consolidation sign was identified, it had high specificity and a high positive predictive value for a diagnosis of DAH. Since the CT imaging features of DAH are varied and hyperdense consolidation is just one of the patterns, the sensitivity was low; however, the high specificity can give confidence to radiologists when they report the possibility of DAH. Other lung diseases that can cause calcification, such as metastatic pulmonary calcification and amyloidosis, may show hyperdense consolidation. However, these are chronic diseases without hemoptysis, and their consolidation may be high even in the normal mediastinal window or bone window setting, whereas CT attenuation value of bleeding does not exceed 100 HU^13,17–19^.

It is important when looking for the hyperdense consolidation sign in a clinical setting to ensure that the CT is not performed with the patient in the arms-down position or if the patient performs poor breath holding. The narrow window setting may emphasize artifacts caused by these conditions and show normal consolidation as hyperdense.

This study had several limitations. First, the window setting may have influenced the identification of hyperdense consolidation sign on CT. The specific narrow window setting in this study (window level = 35 HU, width = 80 HU) was chosen because it is used as the brain window in our hospital. Further consideration may be needed to determine if this setting is optimal. Second, the diagnosis of DAH was made clinically, and there was no pathological confirmation for about half of the patients in our study. BAL could not always be performed because of severe dyspnea and increased risk for hemorrhage. After integration of the patient history, laboratory testing, and imaging findings, we assumed that most of our patients in this study had a high probability of DAH. Third, the small sample size of this study may impair the reliability of our results. Therefore, further studies with larger sample sizes are still required to confirm our results. Finally, the presence of hyperdense consolidation sign was evaluated only by visual assessment, so there was no quantitative evaluation, such as the CT attenuation value with an ROI placement. Since the lung field may contain air even within the consolidation, we suspected that measurement of the CT attenuation value may not be suitable to assess the presence of the hyperdense consolidation sign.

In conclusion, the hyperdense consolidation sign was found to be a highly specific sign for the diagnosis of DAH that can increase the confidence of radiologists when reporting possible DAH.

## Data Availability

The datasets used and/or analysed during the current study available from the corresponding author on reasonable request.
